# Desmoglein-2 F833C Is Predicted to Disrupt Hydrophobic Interactions With the Armadillo Repeat Region 1 of Plakoglobin

**DOI:** 10.7759/cureus.115110

**Published:** 2026-08-24

**Authors:** Vincent Boutsioukos, Eric Johnson

**Affiliations:** 1 Research, Alabama College of Osteopathic Medicine, Dothan, USA; 2 Anatomy, Idaho College of Osteopathic Medicine, Meridian, USA

**Keywords:** arrhythmogenic cardiomyopathy, desmoglein-2, desmosome, missense variant, molecular dynamics, plakoglobin, protein-protein interaction, structural bioinformatics, variant interpretation, variant of uncertain significance

## Abstract

Desmoglein-2 (DSG2) is a desmosomal cadherin involved in cell-cell adhesion within cardiac tissue. A variant of uncertain significance (VUS), involving a phenylalanine-to-cysteine substitution at residue 833 (F833C), was identified and analyzed for its potential impact on the interaction between DSG2 and its intracellular binding partner plakoglobin. Molecular dynamics (MD) simulations, in silico pathogenicity predictions, and structural stability analyses were performed to assess the effects of this substitution. The F833C variant shows significantly reduced binding energy between DSG2 and plakoglobin, along with increased local structural flexibility measured by root mean square fluctuation (RMSF) at the mutation site and surrounding interaction interface. Notably, these local effects were observed despite apparent preservation of the overall global structure of the complex, which may indicate that the pathogenic effect of F833C predominantly involves local destabilization of the binding interface rather than gross conformational change. Structural analysis predicted that the phenylalanine-to-cysteine substitution disrupts hydrophobic interactions between DSG2 and plakoglobin’s armadillo repeat region 1, potentially weakening desmosomal stability. Collectively, these findings suggest that the F833C variant may impair DSG2-plakoglobin interactions, supporting its potential contribution to arrhythmogenic right ventricular cardiomyopathy and warranting further experimental investigation.

## Introduction

Arrhythmogenic right ventricular cardiomyopathy (ARVC) is a progressive inherited cardiac disorder characterized by fibrofatty replacement of ventricular myocardium, ventricular arrhythmias, and an increased risk of sudden cardiac death, particularly in young athletes [1]. While the exact pathophysiology of ARVC continues to be investigated, multiple risk factors for the disease have been proposed in the literature, including family history, male sex, and intense physical activity [1,2]. Most notably, mutations in genes encoding desmosomal proteins are well-established causes of ARVC and are found in approximately 50% of patients with the disease [3].

In the normal, healthy heart, desmosomes serve as intercellular junctions that mechanically couple adjacent cardiomyocytes, allowing the myocardium to withstand the repetitive mechanical stress of cardiac contractions [3]. Desmosomes are composed of transmembrane cadherin proteins (desmoglein-2 (DSG2) and desmocollin-2 (DSC2)) that interact with intracellular plaque proteins (plakoglobin (JUP), plakophilin-2 (PKP2), and desmoplakin (DSP)), which in turn anchor to the intermediate filament cytoskeleton [4]. In patients with ARVC, mutations in desmosomal proteins are thought to compromise the mechanical integrity of these intercellular junctions, leading to progressive cardiomyocyte detachment, cell death, fibrofatty replacement, and ultimately ventricular dysfunction [5].

Mutations in the various genes encoding these desmosomal proteins, including PKP2, DSP, DSG2, DSC2, and JUP, are responsible for a subset of ARVC cases, especially in patients with a family history of the disease [5,6]. DSG2 mutations are commonly associated with ARVC, making up approximately 10% of all genetically confirmed cases [7]. DSG2 is a transmembrane glycoprotein consisting of 1,118 amino acids (AAs) that is primarily found in cardiac desmosomes [8]. The intracellular cadherin segment (ICS) domain (AA 820-843) of DSG2 binds to JUP’s armadillo repeat region, anchoring the desmosome to the intracellular plaque and ultimately to the intermediate filament network [8,9]. Recent structural and biophysical studies have demonstrated that pathogenic variants affecting the desmosomal cadherin-JUP interface alter binding kinetics and weaken intercellular adhesion, supporting the concept that disruption of this interaction is a common molecular mechanism underlying arrhythmogenic cardiomyopathy [10].

Multiple studies on DSG2 variants have supported the conclusion that deleterious mutations in the ICS domain result in weakened desmosomal adhesion and mechanical failure of intercellular junctions, leading to ARVC in thousands of hereditary cardiomyopathy cases [7]. However, there remain multiple variants of uncertain significance (VUS) of DSG2 whose involvement in the pathogenesis of ARVC remains unknown [10]. A variant is classified as a VUS when there is insufficient or conflicting data regarding its pathogenicity, though the growing availability of computational structural modeling and large-scale variant prediction frameworks has substantially improved VUS interpretation, allowing molecular evidence to complement traditional clinical and genetic data when assessing pathogenicity [11,12].

In the case of DSG2, multiple variants in the ICS domain have unknown pathogenicity, including a phenylalanine-to-cysteine variant at position 833 [7,8]. In this brief study, we specifically aimed to evaluate the predicted structural and functional effects of the DSG2 F833C variant through computational analyses, including molecular dynamics simulations, in silico pathogenicity prediction, and structural stability analyses, to assess its potential impact on DSG2-JUP interactions and its possible contribution to ARVC.

## Materials and methods

The FASTA sequences of DSG2 and JUP were accessed on UniProt [8,13], and the Protein Data Bank (PDB) file containing the structure of the E-cadherin/JUP complex was downloaded from the Research Collaboratory for Structural Bioinformatics (RCSB) PDB (PDB identifier: “3IFQ”) [14]. A homology model of the DSG2-JUP complex was constructed by replacing the E-cadherin intracellular domain sequence (residues corresponding to DSG2 AA 820-843) with the DSG2 ICS sequence from UniProt [8]. The JUP armadillo repeat region 1 (AA 126-225) was retained from the original structure [14]. The PDB file was opened in the Yet Another Scientific Artificial Reality Application (YASARA) software [15], and after all water molecules and solvents were removed from the protein complex, a separate file was generated in which phenylalanine at position 833 was replaced with cysteine to construct the F833C DSG2 structure.

The emrunclean.mcr macro was run for the PDB files of the native and F833C variant DSG2-JUP complexes in YASARA Molecular Dynamics (v25.1.13.W.64), generating three replicate PDB files for each complex through energy minimization. Twenty-nanosecond molecular dynamics simulations were performed using the AMBER03 force field [15], with hydrogen bonding and protonation states optimized for pH 7.4 and 0.9% NaCl added to mimic physiological conditions. Bond length and water-angle constraints allowed an intramolecular timestep of 1.333 fs and an intermolecular timestep of 4 fs. Simulations were run at 298 K, and pressure was controlled via simulation-cell rescaling to maintain constant density. Energies for analysis were computed using AMBER14 [15]. Trajectories were analyzed using md_analyze macros to calculate root mean square deviation (RMSD), root mean square fluctuation (RMSF), principal component analysis, hydrogen bonding, solvent-accessible and van der Waals surface areas, per-residue contacts, and secondary-structure content. Simulation trajectories were assessed for equilibration and structural stabilization by examining RMSD and related structural metrics over the course of each simulation. The final 10 ns of each trajectory was selected for comparative analyses after apparent structural stabilization, thereby reducing the influence of initial equilibration on the calculated averages.

Each simulation was independently initialized following YASARA’s molecular dynamics setup and energy-minimization procedures. Because the simulations were performed using YASARA’s standard initialization workflow, no manually specified random seed was applied.

Following the simulation, the average RMSD of the F833C and native replicates across the 20 ns simulations were qualitatively analyzed in Microsoft Excel (Microsoft® Corp., Redmond, WA, USA) to evaluate structural differences in the DSG2-JUP complex. The alpha-carbon RMSD averages of the VUS DSG2 replicates and native replicates from the last 10 ns of the simulations (i.e., after structural stabilization) were calculated, and the datasets were transposed to perform a two-sample t-test assuming equal variances. The radius of gyration was similarly analyzed to assess changes in global compactness, with mean values calculated from the 10-20 ns interval and compared using a two-sample t-test.

The 20 ns simulation duration and three replicates per condition were selected as an exploratory computational sampling strategy to characterize predicted local and global structural effects of the F833C substitution while maintaining computational feasibility. Accordingly, the simulations were not intended to provide exhaustive conformational sampling, and findings were interpreted as predicted molecular effects requiring experimental validation.

Binding energy between DSG2 residues 830-838 and JUP was calculated for both native and F833C complexes using YASARA’s energy-calculation functions [15]. Mean binding energies were determined from each replicate, and statistical significance was assessed via the two-sample t-test assuming equal variances. Local structural dynamics at residue 833 were evaluated by calculating the RMSD and RMSF specifically at this position across all replicates. Per-residue RMSF values were also calculated for the entire DSG2 ICS domain (AA 820-843) and JUP armadillo repeat region 1 (AA 126-225) to identify flexibility changes associated with the F833C mutation. Statistical significance for per-residue comparisons was determined using two-sample t-tests with an alpha threshold of 0.05. The two-sample t-test was selected to compare the mean values between the independent native and F833C replicate groups, with equal variances assumed for the primary comparisons. Given the limited number of independent simulation replicates (n = 3 per condition), statistical results were interpreted as exploratory and were considered alongside the observed structural and molecular dynamics trends rather than as definitive evidence of biological significance.

The PDB identifier of the E-cadherin/JUP complex (3IFQ) was entered into the ConSurf-DB to estimate the evolutionary conservation of residue 833 across species [16,17]. The DSG2 sequence alignment was used to map conservation scores to the corresponding residues [16]. The variant was additionally analyzed through multiple pathogenicity prediction tools, including SIFT [18], PolyPhen-2 [19], and EVE [20], by inputting the DSG2 FASTA sequence and selecting the F833C substitution to predict the likelihood that the F833C substitution is deleterious. Structural analysis of the F833C mutation’s impact on protein-protein interactions was performed using DynaMut2 to identify disrupted residue contacts and predict changes in binding affinity and structural stability [21].

## Results

**Figure 1:**
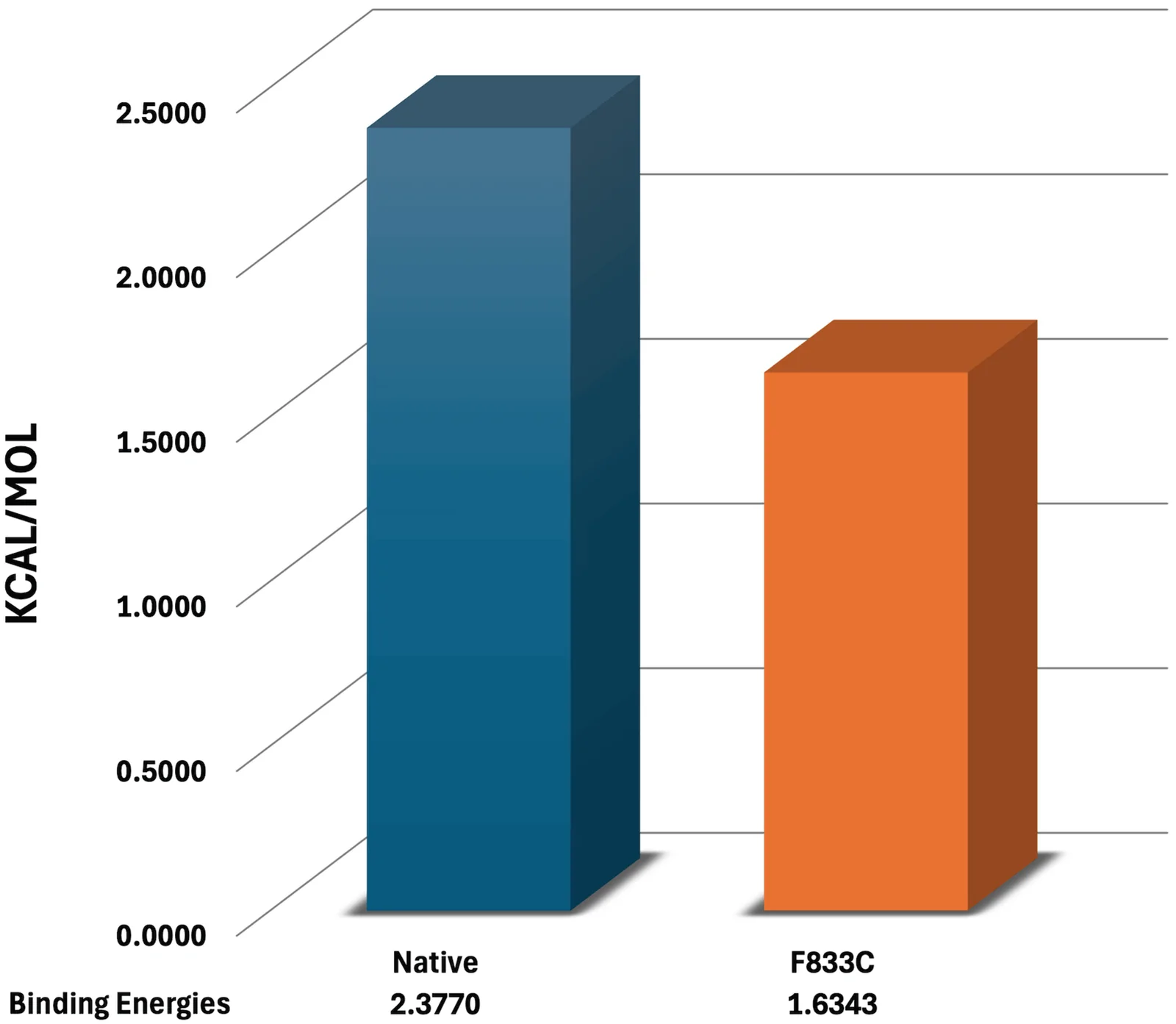
Comparison of Binding Energies Between Native Protein and F833C Mutant Binding energy comparison between native (blue) and F833C variant (orange). The F833C variant shows significantly lower binding energy (p = 0.0009).

The binding energy of the native and F833C variant DSG2-JUP complexes was quantitatively compared to determine whether the F833C mutation caused a significant disruption to the protein-protein interface. A two-sample t-test assuming equal variances was calculated from the binding-energy averages of the variant and native replicates for residues 830-838. As shown in Figure 1, the mean binding energy of the native DSG2-JUP complex was 2.38 kcal/mol, whereas the mean binding energy of the F833C variant was 1.63 kcal/mol. Because the two-tailed p-value (p = 0.0009) was less than the alpha value of 0.05, the difference between the two means was statistically significant, indicating that the F833C substitution substantially reduces the binding affinity between DSG2 and JUP [15].

**Figure 2:**
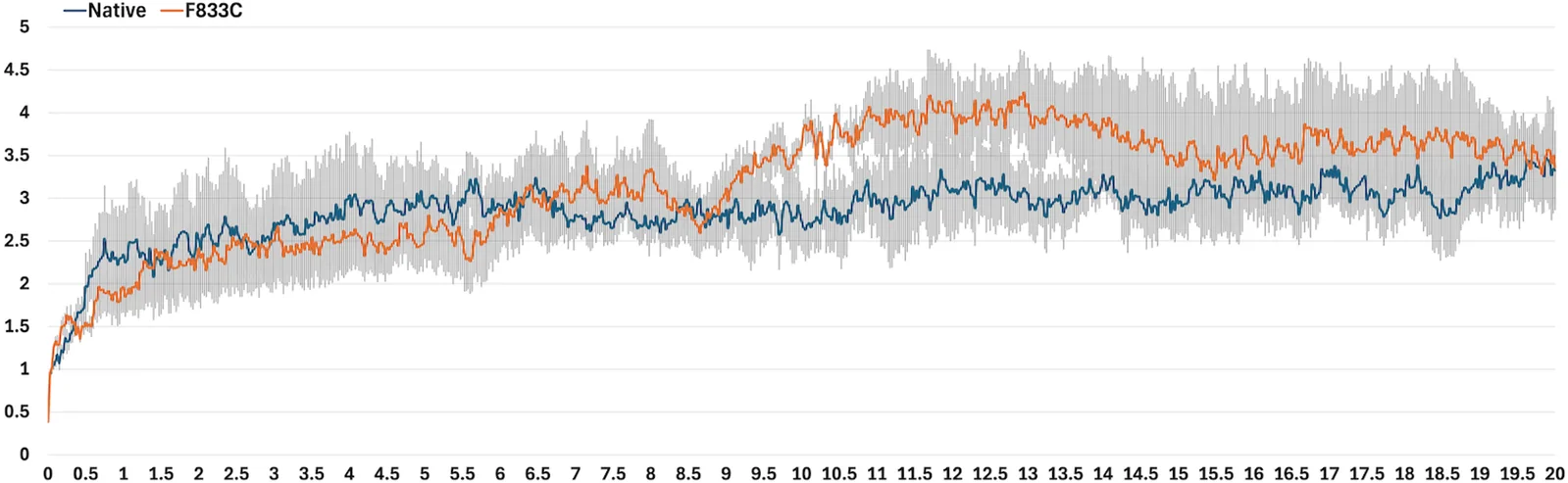
Root Mean Square Deviation (RMSD) Over Time for Native and F833C Variant Structures The graph compares the global RMSD of the native protein (blue) and F833C variant (orange) across the 20 ns simulations. The F833C variant shows a slightly higher mean RMSD (3.72 Å) than native (3.05 Å), but the difference is not statistically significant (p = 0.347).

The RMSD of the native and variant DSG2-JUP complexes across the 20 ns simulations was qualitatively compared to determine whether the F833C mutation caused a significant global structural change. As illustrated in Figure 2, minimal visible differences were observed between the two trajectories, particularly from 10 to 20 ns when the protein conformations had stabilized. A two-sample t-test assuming equal variances was calculated from the global RMSD averages of the last 10 ns. The mean global RMSD value of the native complex was 3.05 Å, while the mean RMSD of the F833C variant was 3.72 Å. Because the two-tailed p-value (p = 0.347) exceeded the alpha value of 0.05, the difference between the two means was not statistically significant [15].

The radius of gyration was analyzed to assess whether the F833C mutation altered the overall compactness of the DSG2-JUP complex. The mean radius of gyration for the native complex was 14.63 Å, whereas the F833C variant exhibited a mean radius of gyration of 14.98 Å. A two-sample t-test revealed that this difference was not statistically significant (t(4) = −1.85, p = 0.138), suggesting that the mutation does not substantially affect global compactness [15].

To investigate local structural dynamics at the mutation site, RMSD and RMSF values at position 833 were analyzed. The mean RMSD at F833 for the native complex was 0.70 Å, whereas the F833C variant showed a significantly elevated mean RMSD of 1.61 Å (p = 0.0051). Similarly, the mean RMSF at position 833 increased from 0.80 Å in the native structure to 1.46 Å in the F833C variant (p = 9.87 × 10⁻⁵). These findings show that the F833C substitution causes significant local destabilization at the mutation site [15].

**Figure 3:**
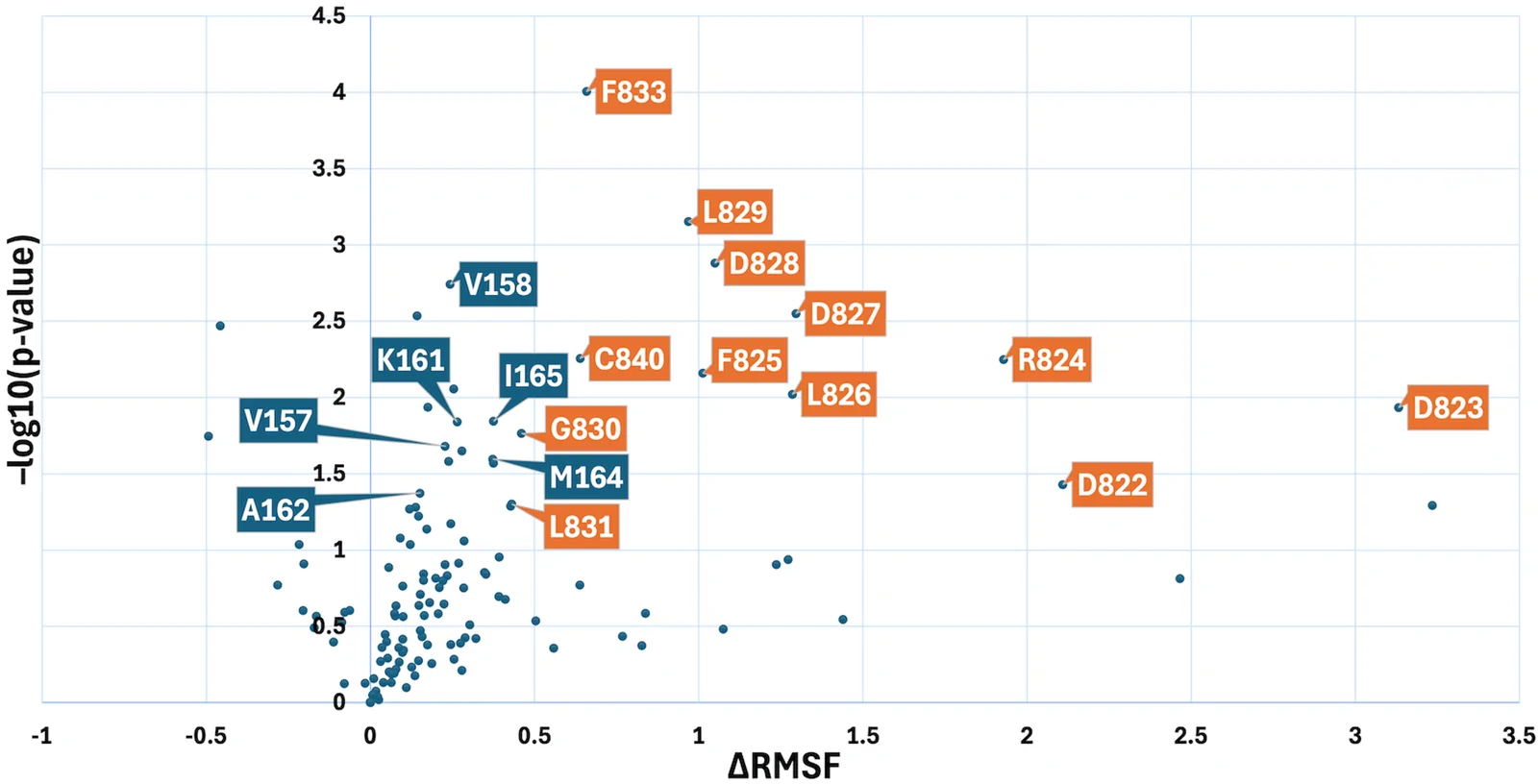
Volcano Plot of Per-Residue RMSF Changes at the DSG2-Plakoglobin Interface of F833C The plot compares the magnitude of flexibility change (ΔRMSF, x-axis) with statistical significance (-log10(p-value), y-axis). Orange labels denote DSG2 residues (D822-C840) and blue labels denote plakoglobin residues (V157-I165), showing statistically significant increases in flexibility (p < 0.05). Notably, the mutation site F833 demonstrated a significant RMSF increase in the F833C variant compared to native (1.46 Å vs. 0.80 Å, p = 9.87 × 10⁻⁵). RMSF: root mean square fluctuation

Per-residue RMSF analysis was performed to identify regions of altered flexibility at the DSG2-JUP interface. As shown in Figure 3, several DSG2 residues exhibited significantly increased flexibility in the variant structure. Specifically, residues D822 through L831, F833, and C840 show statistically significant increases in RMSF (p < 0.05), indicating enhanced local mobility within the ICS. On the JUP side of the interface, residues V157, V158, K161, A162, M164, and I165 within armadillo repeat region 1 also exhibited significantly elevated RMSF values (p < 0.05), suggesting destabilization of the complementary binding surface.

The F833C mutation was evaluated using multiple pathogenicity-prediction tools. As shown in Table 1, ConSurf indicates that residue 833 is highly evolutionarily conserved, whereas EVE, SIFT, and PolyPhen-2 predicted the F833C variant to be deleterious or pathogenic. ConSurf assigned a conservation score of nine, indicating strong evolutionary constraint [16,17]. EVE predicted the variant as pathogenic with high confidence [20], SIFT classified it as deleterious [18], and PolyPhen-2 predicted it to be probably damaging [19]. Collectively, these computational analyses suggest that the F833C substitution is likely to have deleterious functional effects and support its potential pathogenicity.

**Table 1:** In Silico Pathogenicity and Conservation Analysis of the F833C Variant Summary of bioinformatic prediction tools evaluating the functional impact and evolutionary conservation of the F833C variant. Analyses were conducted using ConSurf, Evolutionary Variant Effect (EVE), SIFT, and PolyPhen-2. High conservation scores and consistent predictions of pathogenicity across multiple independent algorithms support the deleterious structural impact of the variant.

Program	Result	Score/classification
ConSurf	Highly conserved	Conservation score: 9
EVE	Pathogenic	High confidence
SIFT	Deleterious	Score 0.05
PolyPhen-2	Probably damaging	Score > 0.85

## Discussion

In the current study, we analyzed a VUS of DSG2 to determine its potential effect on the pathogenesis of ARVC. As mentioned, mutations in the ICS of DSG2 cause disruption of desmosomal integrity within cardiac tissue, leading to mechanical instability during cardiac contractions and progressive cardiomyocyte loss characteristic of ARVC [5,7]. We selected for study a phenylalanine-to-cysteine mutation at residue 833, a variant located within the critical ICS domain that mediates the interaction between DSG2 and JUP [4,9]. To comprehensively characterize the effects of the F833C variant, we performed quantitative and qualitative analyses of binding energies, global RMSD, radius of gyration, and local RMSD/RMSF of the native DSG2-JUP complex and variant complex, production of per-residue RMSF profiles, analysis of the evolutionary conservation patterns of native DSG2 with the ConSurf-DB program, and examination of collective predictions of the DSG2 mutation through multiple pathogenicity prediction tools [15,16,18-21].

The simulation results were compared via RMSD graphs of the native and variant complexes across the simulation [15]. As demonstrated in Figure 2, both trends were nearly identical on qualitative analysis, consistent with the absence of large-scale structural changes in the complex that might have been anticipated by the mutation. No visible differences are recognized between the two graphs, especially from 10 to 20 ns, when the protein conformations had stabilized. These findings altogether represent a minimal apparent impact of the F833C substitution on the global structure of the DSG2-JUP complex. We also calculated the RMSD averages of the F833C DSG2 replicates and the native replicates of the last 10 ns of the simulations to produce a two-sample t-test for quantitative analysis of possible global structural changes [15]. The p-value was greater than the alpha value of 0.05 (p = 0.347), indicating no statistically significant difference between the RMSD averages of the two complexes, therefore no significant difference in the overall native and variant structures. Similarly, the radius of gyration analysis revealed no significant change in the global compactness of the complex (p = 0.138), further supporting the conclusion that the F833C mutation does not induce large-scale conformational changes [15]. This interpretation is consistent with prior work on desmosomal proteins. In a structural review of representative buried, semi-buried, and surface-exposed missense mutations, Mohammed and Chidgey illustrated how such variants can alter the structural fold of cardiac desmosomal proteins and thereby compromise desmosomal adhesion and cause ARVC, rather than acting predominantly through global protein destabilization [9]. Zhang et al. similarly emphasized that, although desmosome dysfunction is a common pathogenic pathway in DSG2-related arrhythmogenic cardiomyopathy, the molecular mechanisms underlying this dysfunction vary among individual DSG2 mutations, with downstream consequences including JUP dislocation and gap junction dysfunction [7]. Our finding that F833C reduces predicted DSG2-JUP binding affinity and increases local interface flexibility while preserving the global structure of the complex is therefore consistent with a local, interface-level mechanism of the kind these reviews describe. To our knowledge, no prior study has experimentally characterized the DSG2 F833C variant specifically.

The preserved global structure initially appears to contrast with the results of the binding energy analysis, where the F833C mutation was shown to significantly reduce the binding affinity between DSG2 and JUP [15]. The mean binding energy decreased from 2.38 kcal/mol in the native complex to 1.63 kcal/mol in the F833C variant (p = 0.0009), representing a 31.2% reduction in binding strength (Figure 1). This substantial decrease in binding energy suggests that the F833C substitution may compromise the stability of the DSG2-JUP interface despite preserving the overall global structure of the complex. The discrepancy between preserved global structure and reduced binding affinity indicates that the pathogenic effect of F833C may operate through local destabilization rather than gross conformational changes.

This interpretation is strongly supported by the local RMSD and RMSF analyses at position 833 [15]. The F833C variant exhibited significantly elevated RMSD (1.61 Å vs. 0.70 Å, p = 0.0051) and RMSF (1.46 Å vs. 0.80 Å, p < 0.0001) at the mutation site compared to native. These metrics indicate that while the global structure remains intact, the local environment at position 833 experiences substantial instability and increased flexibility. This local destabilization may account for the reduced binding energy observed between DSG2 and JUP, as the mutation disrupts critical interactions at the interface without causing the entire complex to unfold or dramatically reorganize.

The per-residue RMSF analysis provides further mechanistic insight into how the F833C mutation destabilizes the DSG2-JUP interface [15]. As shown in Figure 3, the mutation produced a cascade of increased flexibility extending beyond the immediate mutation site. On the DSG2 side, residues D822 through L831, F833, and C840 all show significantly elevated RMSF values (p < 0.05), indicating that the F833C substitution destabilizes a substantial portion of the ICS domain. This extended region of increased flexibility suggests that the mutation disrupts a network of stabilizing interactions rather than affecting only a single contact point.

**Figure 4:**
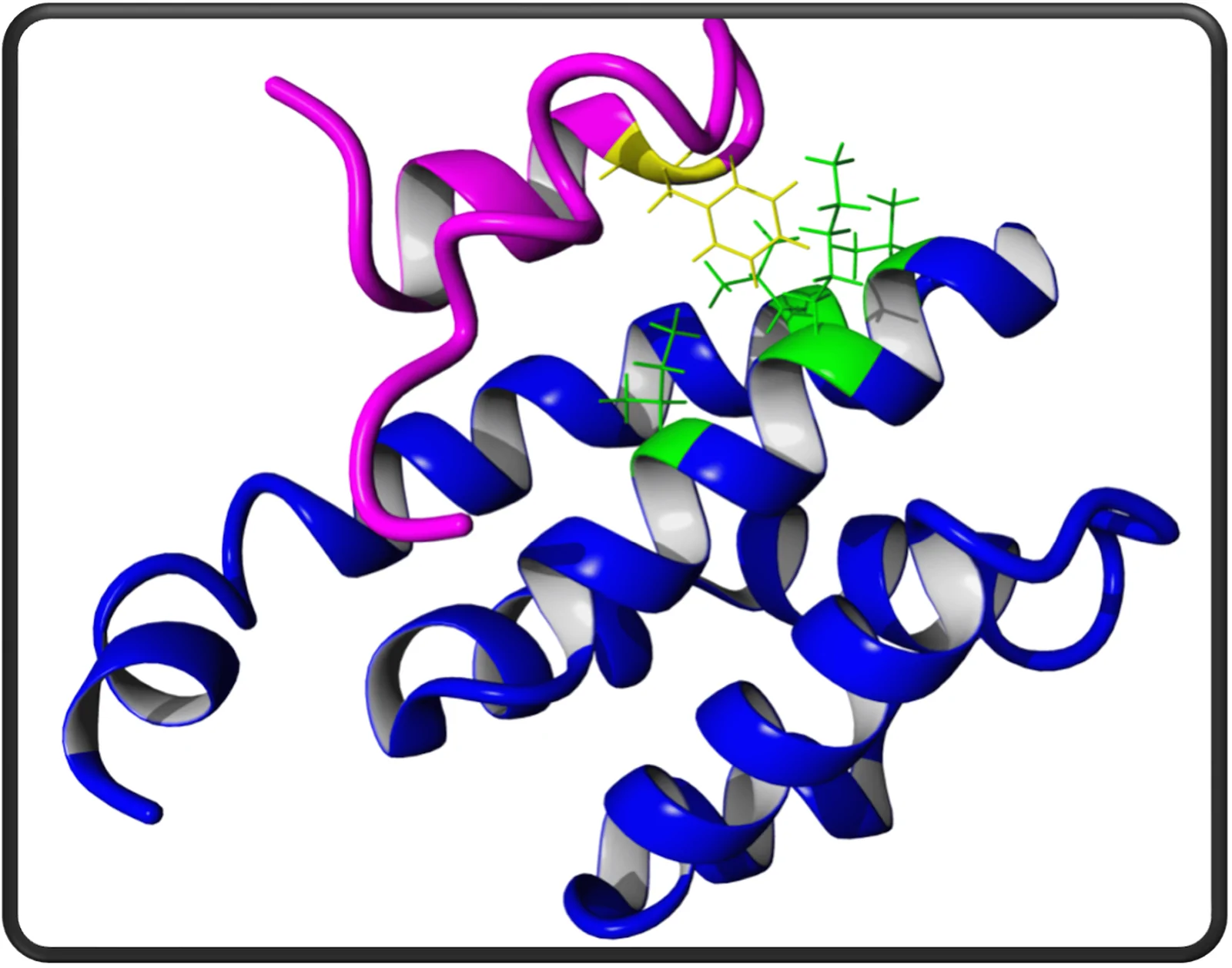
Structural Model of the DSG2-Plakoglobin Complex Predicted structural interactions of DSG2 F833 with plakoglobin armadillo repeat region 1 are shown, with hydrophobic interactions identified by DynaMut2 [21]. A three-dimensional ribbon representation of the DSG2-plakoglobin interaction interface depicts DSG2 in magenta/yellow and plakoglobin in blue/green. The phenylalanine residue at position 833 (F833) on DSG2 is highlighted in yellow, while plakoglobin residues L150, V158, K161, and I165 are highlighted in green, illustrating the key hydrophobic interaction pocket at the binding interface.

Critically, the F833C mutation also significantly increases the flexibility of multiple residues within JUP's armadillo repeat region 1, specifically V157, V158, K161, A162, M164, and I165 (p < 0.05) [15]. According to structural modeling with DynaMut2, the native phenylalanine at position 833 forms hydrophobic interactions with L150, V158, K161, and I165 on JUP (Figure 4) [21]. The substitution of phenylalanine with cysteine eliminates these critical hydrophobic contacts, as cysteine's smaller side chain and polar thiol group cannot maintain the same interaction network [21]. The loss of these stabilizing hydrophobic interactions may explain both the reduced binding energy and the increased flexibility observed in JUP's armadillo repeat 1 residues. This finding is particularly noteworthy because armadillo repeat 1 represents an important binding surface for desmosomal cadherins, and its destabilization would be expected to compromise desmosome function [4,6,14].

The pathogenic relevance of disrupting interactions with JUP's armadillo repeat 1 is supported by previous studies of ARVC-associated mutations [4,7,9]. Ly et al. reported a V159L mutation in JUP that causes ARVC, and this residue lies directly within the region affected by the F833C mutation (residues 157-165) [6]. The fact that mutations on either side of the interface, whether in DSG2 (F833C) or in JUP (V159L), can cause ARVC strongly suggests that the integrity of this specific protein-protein interaction is critical for desmosomal function and cardiac mechanical stability [4,6,9]. The F833C mutation likely produces a similar pathogenic effect to the V159L mutation by disrupting the same interaction surface from the opposite side of the interface. Beyond its structural role in desmosomes, JUP also functions as a signaling molecule that modulates Wnt/β-catenin transcriptional activity, and disruption of the DSG2-JUP interface may therefore have downstream effects on gene expression pathways implicated in the fibrofatty replacement characteristic of ARVC [22].

The ConSurf-DB program was utilized to examine the evolutionary conservation of DSG2, specifically the phenylalanine residue at position 833 [16,17]. Highly conserved residues evolve at slower rates and are generally located within regions essential for protein structure, ligand binding, or function; substitutions at these positions are therefore more likely to be pathogenic [16,17]. As seen in Table 1, the phenylalanine residue at 833 received a conservation score of nine, the highest possible, indicating strong evolutionary constraint and suggesting that the F833C substitution is likely to be deleterious to DSG2 function or to the stability of the protein-protein interface [16,17].

Multiple pathogenicity prediction tools were used to estimate the likelihood of the DSG2 F833C substitution being disease-causing. Table 1 demonstrates the consistent prediction of F833C to be a deleterious or pathogenic mutation across all programs [18-20]. EVE predicted the variant as pathogenic with high confidence [20], SIFT classified it as deleterious [18], and PolyPhen-2 predicted it to be probably damaging [19]. The agreement among these computational prediction tools supports the hypothesis that the F833C substitution may have deleterious functional effects. However, computational pathogenicity prediction tools have inherent limitations and should not be interpreted as definitive evidence of disease causality. These tools rely on sequence conservation, evolutionary models, structural features, or other computational assumptions and may produce concordant predictions without independently establishing a biological or clinical phenotype. Therefore, the agreement among prediction tools in this study should be considered supportive evidence rather than confirmation of pathogenicity. These findings correlate with those of the binding energy analysis, local RMSD/RMSF analysis, per-residue RMSF profiles, and ConSurf-DB study, which collectively suggest that the F833C substitution may have molecular effects that could contribute to ARVC pathogenesis by reducing the stability and binding affinity of the DSG2-JUP interface. Experimental validation using binding assays, cellular desmosome assembly studies, and clinical correlation in variant carriers will be necessary to confirm these predicted molecular effects and their contribution to ARVC pathogenesis.

A potential mechanism by which the F833C mutation may contribute to ARVC can be understood through the integration of our structural and dynamic findings. Desmosomes function as mechanical anchors that connect the intermediate filament cytoskeleton of adjacent cardiomyocytes, allowing the heart to withstand the repetitive mechanical stress of cardiac contractions [10]. The DSG2-JUP interaction is a critical component of this mechanical linkage, as JUP serves as an adaptor protein connecting desmosomal cadherins like DSG2 to the intracellular desmosomal plaque proteins [22]. The computational analyses suggest that the F833C mutation may weaken this connection by reducing binding energy and increasing local flexibility at the interface, disrupting the DSG2-JUP interface through loss of critical hydrophobic interactions [4,10]. This proposed mechanism is consistent with the "common pathway" hypothesis of ARVC pathogenesis, which posits that mutations in any of the desmosomal proteins (DSG2, JUP, PKP2, DSP, and DSC2) ultimately lead to the same disease phenotype by compromising mechanical integrity [1,23]. Over time, the cumulative effect of cardiac mechanical stress on a potentially weakened desmosome could contribute to progressive mechanical failure, leading to cardiomyocyte detachment, fibrofatty replacement, and the characteristic pathology of ARVC [5]. The fact that the mutation causes local rather than global structural changes may provide one possible explanation for why ARVC often has variable penetrance and age-dependent onset, as weakened desmosomes may function adequately under normal conditions but fail progressively under the cumulative mechanical stress of years of cardiac activity [10,23].

The primary limitation of our study is the use of a homology model based on the E-cadherin/JUP complex structure rather than a direct crystal structure of the DSG2/JUP complex. While E-cadherin and DSG2 share significant sequence homology in their intracellular domains and both interact with JUP through similar mechanisms, subtle differences in the binding interfaces may exist that are not captured in our model. However, the high degree of conservation of the ICS domain across classical cadherins and desmosomal cadherins, combined with the consistent results across multiple analytical approaches, suggests that our homology model provides a reasonable approximation of the native DSG2-JUP interaction. Furthermore, we did not analyze the interaction of the DSG2 F833C variant with other desmosomal proteins such as PKP2 or DSP. Although the F833C substitution significantly affected the DSG2-JUP interface, there remains the possibility that the mutation may also alter DSG2's interactions with other components of the desmosomal plaque or induce compensatory changes in the overall desmosome structure. Another limitation of our study is the relatively short simulation time of 20 ns and the small sample size of three replicates for each structure. While our simulations were sufficient to observe equilibration and detect significant differences in binding energy and local flexibility, longer simulations might reveal additional conformational changes or rare events that occur on slower timescales. The limited number of simulation replicates also reduces statistical power and increases uncertainty in estimates of between-group differences. Accordingly, the reported p-values should be interpreted cautiously, particularly given the small number of independent simulation replicates. To address these limitations, future computational work should combine longer molecular dynamics simulations with larger numbers of replicates and enhanced sampling to more fully characterize the conformational landscape, and should extend the analysis to DSG2's interactions with other desmosomal plaque components such as PKP2 and DSP. Complementary experimental validation of the variant's effect on DSG2-JUP binding and desmosomal function, as described in the Conclusion, would then test whether these predicted molecular effects translate into measurable adhesive and mechanical deficits. Importantly, the computational findings presented here should not be interpreted as sufficient to establish pathogenicity or support clinical reclassification of F833C. Functional studies demonstrating an effect on DSG2-JUP binding or desmosomal function, together with clinical and genetic evidence from variant carriers, will be necessary before the variant can be considered for reclassification from a VUS.

## Conclusions

Based on the findings of our study, we conclude that the DSG2 F833C substitution is predicted to alter the DSG2-JUP interface and may have functional consequences relevant to ARVC. Analysis across multiple computational approaches suggests that the F833C variant may reduce binding energy, increase local flexibility, disrupt critical hydrophobic interactions with JUP's armadillo repeat 1, and affect a highly evolutionarily conserved residue. The mutation is predicted to operate primarily through local interface destabilization rather than global structural disruption, potentially weakening the mechanical linkage between DSG2 and JUP that is essential for desmosomal integrity during cardiac contractions. However, because these findings are based exclusively on computational analyses, they should be considered hypothesis-generating rather than definitive evidence of pathogenicity. Functional studies and clinical evidence are required to determine whether these predicted molecular effects translate into impaired desmosomal function and an ARVC phenotype. Accordingly, the F833C variant should remain a VUS until sufficient functional and clinical evidence is available to support any potential reclassification. As a direct next step, these computational predictions should be tested experimentally: co-immunoprecipitation and surface-based binding assays could confirm the predicted reduction in DSG2-JUP binding affinity, cellular localization and dispase-based cell dissociation assays could assess whether the variant impairs desmosomal assembly and adhesive strength, and atomic force microscopy could quantify the mechanical consequences of the substitution at the desmosome. Clinical correlation in carriers of F833C would then help establish whether these molecular effects translate into an ARVC phenotype. These studies would help determine whether the predicted structural effects have functional and clinical significance and whether the variant warrants future consideration for reclassification. We hope that our findings will encourage further investigation of this and other DSG2 variants, ultimately contributing to enhanced diagnostics, genetic counseling, and targeted therapies for patients with hereditary ARVC.
